# Ameliorating a vertical axis wind turbine performance utilizing a time-varying force plasma actuator

**DOI:** 10.1038/s41598-024-69455-8

**Published:** 2024-08-08

**Authors:** Sarallah Abbasi, Mohammad Amin Daraee

**Affiliations:** Mechanical Engineering Department, Arak University of Technology, Arak, Iran

**Keywords:** Wind energy, VAWT, Plasma actuator, Time-varying force, Energy science and technology, Engineering, Mathematics and computing, Physics

## Abstract

Controlling wind flow on vertical axis wind turbine blades is an effective technique for enhancing their performance. Modern equipment such as plasma actuators have gained significant attention for their ability to control, and improve the flow behavior in wind turbines. Previous studies have primarily focused on investigating plasma actuators with constant force. In this study, plasma actuators with varying forces over time were applied to the turbine blades. An unsteady 2D model was used to analyze the wind turbine. The sliding mesh model was employed to simulate rotor rotation, and the SST $k-\omega$ model was utilized for turbulence modeling. Initially, the performance of the clean turbine was examined. In the next step, the plasma actuators with different force waveforms were applied to the wind turbine blades, including constant, sine, cosine, positive ramp, negative ramp, pulse in the first half-cycle, and pulse in the last half-cycle waveforms. The results indicated that the cosine, and sinusoidal waveforms, led to the greatest improvement with 37.28% and 35.59% increase in the net energy produced by the turbine, respectively, compared to the baseline case.

## Introduction

Increasing power extraction from renewable energy sources is crucial for achieving socio-economic development^1^. By reducing reliance on fossil fuels, the long-term sustainability of the global economy is ensured while mitigating environmental degradation^2,3^. Wind energy, which is considered as an indirect manifestation of solar energy, is recognized an Inexpensive, sustainable, accessible, and eco-friendly option^4^. Increasing the performance, and improving the defects of wind turbines as wind energy exploitation tools is of great importance. Vertical axis wind turbines (VAWTs) are appropriate options for urban areas because of their omnidirectionality, cost-effectiveness, and ability to operate in low-altitude regions with low wind speeds. Despite their advantages over horizontal axis turbines, some weaknesses of VAWTs reduce their efficiency^5–7^. Most of these weaknesses are attributed to flow separation, and the formation of vortices on the blades. Over the past few decades, various approaches were studied with the aim of ameliorating the flow behavior on their blades^8,9^.

Among these methods, plasma actuators have emerged as efficient tools for controlling, and improving the flow behavior on wind turbine blades. A plasma actuator consists of two metal electrodes, and a dielectric, creating an electric field between the two electrodes when voltage is applied. The actuator is installed on the blade surface, and the electric field ionizes the airflow in the boundary layer. This ionized air is accelerated by the electric field and its momentum increases. By increasing the momentum of the airflow in the boundary layer, flow separation is reduced, and vortices are suppressed^10,11^. Over the past few years, some studies have investigated the influence of plasma actuators on VAWTs. Greenblatt et al.^12^ experimentally examined a 2-blade VAWT, finding that installing the actuator on the leading edge of blades increased the power generated by VAWT by 38%. Ma et al.^13^ examined the impact of different plasma actuator activity timings on a straight-blade VAWT, aiming to identify the optimal actuator activity interval with the lowest power consumption. In the optimal case, the torque coefficient increased by 34.3%. Benmoussa et al.^14^ demonstrated an 11% lift increase by applying a plasma actuator to the blades of a 6-blade cyclorotor. In another study by Benmoussa et al.^15^, the influence of utilizing multiple plasma actuators on cyclorotor performance was explored. The findings exhibited a notable increase of 2.3% in thrust, and a corresponding reduction of 0.9% in actuator power consumption. Furthermore, another study conducted by the same researchers demonstrated substantial advancements in the power coefficient of a self-pitch cyclorotor when equipped with a plasma actuator^16^. Xu et al.^17^ revealed that enhancing the voltage of the plasma actuator is able to ameliorate a Savonius wind turbine power coefficient by 43.836%. Jafari et al.^18^ examined the influence of the plasma actuator voltage on a VAWT performance. Their findings showed that the power coefficient of VAWT increases with enhancing the voltage of the actuator at low speeds of free wind flow. Chavoshi et al.^19^ demonstrated a 10% increase in VAWT output power by installing plasma actuators near the leading edge of the blades.

The aforementioned studies mainly focused on using actuators with a constant force over time. While the results of some other studies showed that applying the force caused by the plasma actuator in a time-varying manner can improve the efficiency of the actuator more significantly. Benard et al.^20^ examined the impact of different voltage waveforms of the actuator mounted on a flat plate. They examined sine, square, positive ramp, and negative ramp waveforms, and found that the square waveform yielded optimal body force, while the sine waveform led to increased speed fluctuations. Nakano et al.^21^ studied the effects of voltage waveform on plasma actuator performance. They analyzed triangular, sinusoidal, and square waveforms, and discovered that negative steep-gradual waveforms generated stronger body force. They also observed a 25.1% performance improvement with a negative ramp waveform compared to a sinusoidal waveform. Pescini et al.^22^ optimized the plasma actuator excitation waveform for controlling flow separation in a low-pressure turbine. They compared the results of square, triangular, and sinusoidal waveforms with the case without actuator. The findings indicated that the sinusoidal waveform effectively controlled the flow separation, and increased the torque coefficient compared to the square waveform, although the power lost by the actuator in the square wave was 25.5% higher than the sinusoidal. Alexandre et al.^23^ optimized the applied voltage waveform of a plasma actuator, suggesting nanosecond-pulses of voltage as the optimal waveform. Their findings indicated that this waveform induced higher fluid velocities compared to the conventional sine waveform. Konstantinidis et al.^24^ in a review study, investigated the flow control of bluff bodies using plasma actuators from various perspectives. They explored the effects of configuration, actuator position, excitation frequency, etc. The results highlighted the possibility of increasing electromechanical efficiency by optimizing the input waveform. Matsunuma et al.^25^ experimentally investigated the effects of a non-continuous voltage signal of the plasma actuator on the reduction of vortices on a turbine cascade. Unlike continuous signals, the investigated signal consisted of two parts; the operation sinusoidal part, and non-operation part. The results showed that increasing the operation time to 50% of the total time of a period causes a 38% increase in the peak velocity. Mazaheri et al.^26^ conducted a study to examine the influence of various voltage waveforms on the aerodynamic performance of a wind turbine airfoil. The airfoil was modeled individually, and an electrostatic model was improved to numerically model the actuator. Their findings unveiled a notable advantage of the rectangular waveform over both sinusoidal, and triangular waveforms in terms of augmenting the airfoil lift coefficient.

In Table 1, a summary of the research review is presented. The literature review highlights the effectiveness of plasma actuator as one of the modern tools for ameliorating the flow behavior on wind turbines. The simplicity, small dimensions, low cost, and light weight of this equipment caused the attention of many researchers to be attracted in recent years. Previous research mainly examined the influences of the number and arrangement of actuators, and most used a constant force approach, and so far, no study has been conducted with the aim of investigating a plasma actuator with a time-varying force. The main motivation of this paper is examining the impact of using actuator with variable force. In this study, different waveforms such as constant, pulsed, linear, sinusoidal, and cosine are considered for the plasma actuator force, and their effects on improving the turbine performance is investigated.

**Table 1:** Summary of research review.

Study	Year	Method	Relative increase in performance (%)
Greenblatt et al.^12^	2012	Experimental	38
Matsunuma et al.^25^	2015	Experimental	38
Pescini et al.^22^	2018	Numerical	25.5
Ma et al.^13^	2019	Numerical	34.3
Nakano et al.^21^	2019	Numerical	25.1
Benmoussa et al.^14^	2021	Numerical	11
Xu et al.^17^	2020	Numerical	43.836
Jafari et al.^18^	2022	Experimental	28
Chavoshi et al.^19^	2022	Numerical	10

## Equations governing the problem

### VAWT performance equations

**Figure 1:**
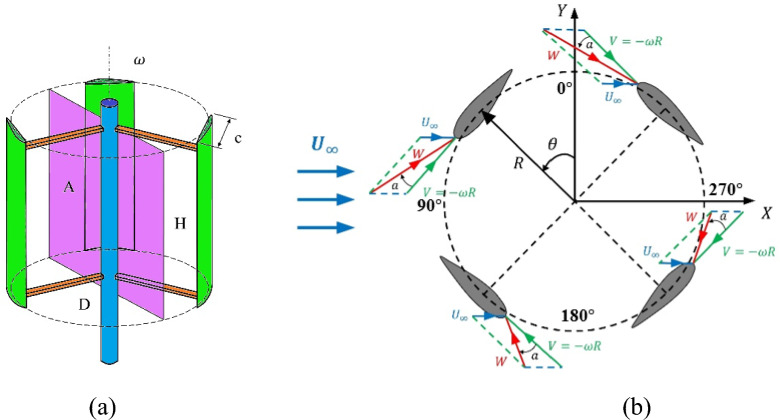
Velocity vectors acting on VAWT blades.

Figure 1 shows the velocity vectors on the VAWT blades at different azimuth angles ($\theta$). ${\overrightarrow{U}}_{\infty }$, $\overrightarrow{V}$, $\overrightarrow{W}$, and $\alpha$ represent the wind flow velocity, the negative linear velocity vector, the relative velocity vector, and angle of attack, respectively. In order to better understand the velocity triangles and avoid the complexity of the figure, the wake effect is not included in this figure. Equations (1), (2) can be used to calculate $\overrightarrow{W}$, and $\alpha$ in different azimuths^27^.

1 $$W={U}_{\infty }\sqrt{{\lambda }^{2}+2\lambda cos\theta +1,}$$

2 $$\alpha ={tan}^{-1}\left(\frac{sin\theta }{\lambda +cos\theta }\right).$$

The following parameters are considered to evaluate the turbine performance^28,29^:

Tip speed ratio (TSR):

3 $$\lambda =\frac{\omega R}{{U}_{\infty }}.$$

Torque coefficient:

4 $${C}_{m}=\frac{M}{\frac{1}{2}\rho {A}_{S}{U}_{\infty }^{2}R}.$$

Power coefficient:

5 $${C}_{p}=\frac{P}{\frac{1}{2}\rho {A}_{S}{U}_{\infty }^{3}}=\lambda {C}_{m},$$

where $\omega$ denotes the rotor angular velocity [rad/s], $R$ is the radius of the rotor [m], ${U}_{\infty }$ is the incoming wind velocity [m/s], *M* is torque [N.m], *P* is output power, $\rho$ is the air density [kg/m^3^], and ${A}_{S}$ is the swept area [m^2^].

### Flow field equations

The unsteady Reynolds-averaged Navier-Stokes (URANS) equations were used to simulate the flow field, assuming 2D unsteady, incompressible, and turbulent. The SST k-ω turbulence model which has been widely used in previous studies^30,31^, was utilized for simulating the turbulent flow on airfoils. Ignoring the energy equation, the governing equations of the flow field are defined by Eqs. (6) and (7).

6 $$\frac{\partial {\overline{u} }_{i}}{\partial {x}_{i}}=0,$$

7 $$\frac{\partial {\overline{u} }_{i}}{\partial t}+{\overline{u} }_{j}\frac{\partial {\overline{u} }_{i}}{\partial {x}_{j}}=-\frac{1}{\rho }\frac{\partial \overline{p}}{\partial {x }_{i}}+\nu \frac{{\partial }^{2}{\overline{u} }_{i}}{\partial {x}_{j}\partial {x}_{j}}-\frac{\partial (\overline{{u }_{i}{\prime}{u}_{j}{\prime}})}{\partial {x}_{j}}+{F}_{b},$$

where $\rho$ is the air density, $\upnu$ is kinematic viscosity, ${F}_{b}$ is the force of the plasma actuator, $\overline{p }$ is time-average pressure. ${\overline{u} }_{i}$ and ${u}_{i}{\prime}$ are the mean and fluctuating components of the velocity, and $\overline{{u }_{i}{\prime}{u}_{j}{\prime}}$ denotes the Reynolds stress tensor. ${x}_{i}$ and ${x}_{j}$ represent the direction of the flow, and the direction perpendicular to the flow, respectively^32^.

### Numerical method to model the plasma actuator

**Figure 2:**
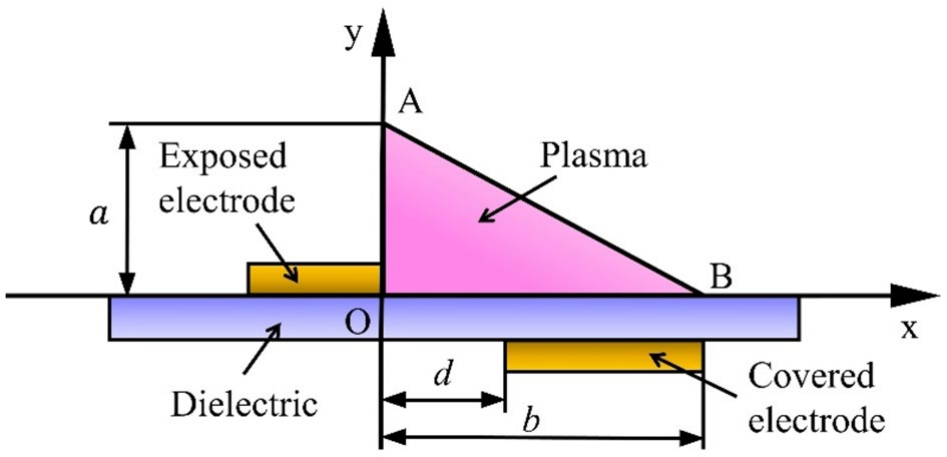
Schematic of a plasma actuator.

The well-known model presented by Shyy et al.^33^ was employed to model the forces caused by the plasma actuator. This numerical model simplifies the electric field of the plasma actuator as a linear field in a triangular region. Installing the plasma actuator on the blade, and inducing force to the ionized the air of the boundary layer flow increases the momentum, and velocity of the boundary layer, and prevent flow separation. Figure 2 shows a schematic of a simple plasma actuator.

The electric field distribution is defined according to Eq. (8).

8 $$E={E}_{0}-{k}_{1}x-{k}_{2}y,$$

where ${E}_{0}$ denotes the electric field at distance d between electrodes, and it can be calculated according to Eq. (9).

9 $${E}_{0}=\frac{{U}_{0}}{d},$$

where ${U}_{0}$ is the voltage applied to the actuator.

${k}_{1}$ and ${k}_{2}$ are defined as Eqs. (10) and (11) where, ${E}_{cr}$ represents the electric field at the interface between plasma, and fluid regions. $a$ and $b$ represent the height, and length of the triangular area, respectively.

10 $${k}_{1}=\frac{{(E}_{0-}{E}_{cr})}{b},$$

11 $${k}_{2}=\frac{{(E}_{0-}{E}_{cr})}{a}.$$

The plasma actuator body force distribution is defined as Eq. (12).

12 $$F=\upsilon \alpha {\rho }_{c}e\Delta t\delta E,$$

where, $\upsilon$, $\alpha$, ${\rho }_{c}$, $e$, $\Delta t$, and $\delta$ represent the frequency of the voltage applied to the actuator, the particle collision efficiency factor, the density of the charge, the charge of the electron, the discharge time of the plasma in one excitation cycle, and the delta function (defined as 1 in plasma activity areas and 0 outside of it), respectively. More details and parameters are provided in references^33^.

## Analysis method

### 2D model and solver settings

**Figure 3:**
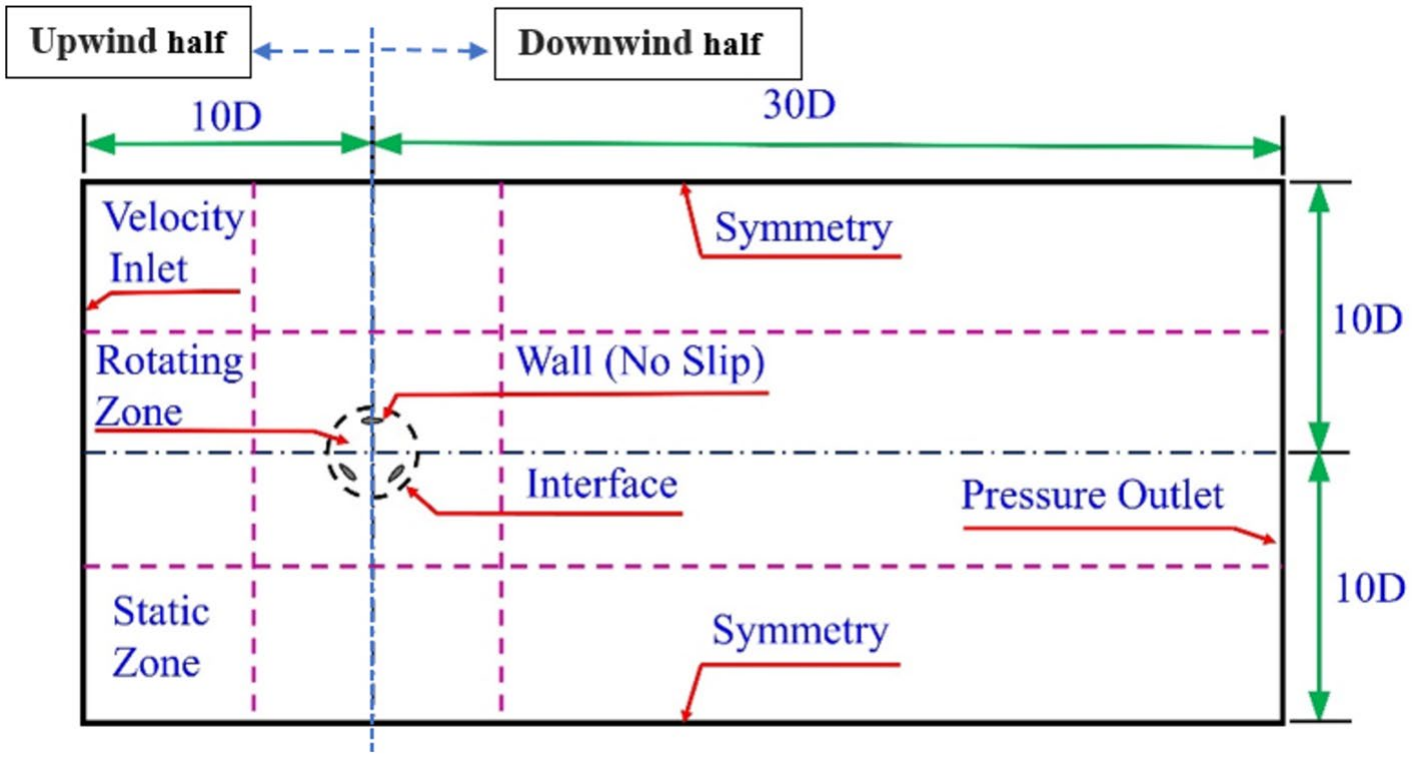
Computational domain and boundary conditions.

The VAWT investigated in this study was modeled according to the turbine dimensions, and operating conditions described in the study of Howell et al. Figure 3 depicts a schematic of the computational area along with boundary conditions. The design of the computational domain was as a rectangular area with dimensions of 40 times the rotor diameter in length, and 20 times the rotor diameter in width, denoted as D. The dimensions of the computational domain were carefully selected to ensure preventing reverse flow and inaccurate outcomes due to improper demarcation. Consequently, the analysis results remained unaffected by the size of the domain. The computational area consists in the rotating and the static parts. To model the rotation of the rotor, a sliding mesh model was employed.

Two-dimensional simulations were conducted using the ANSYS FLUENT software, a commercial computational fluid dynamics (CFD) code. For the pressure–velocity coupling, the well-established SIMPLE algorithm (semi-implicit method for pressure linked equation) was employed. The least-square cell-based approach was utilized for the gradient of the solution variable. For the momentum, turbulent kinetic energy, and specific dissipation rate, the second-order upwind scheme was employed^34^. Unsteady and incompressible conditions were used for the flow, where the air density and viscosity were considered to be equal to 1.225 (kg/m^3^) and $1.7894\times {10}^{-5}$(kg/m.s), respectively^35^.

### Analysis independence from mesh and time step

**Figure 4:**
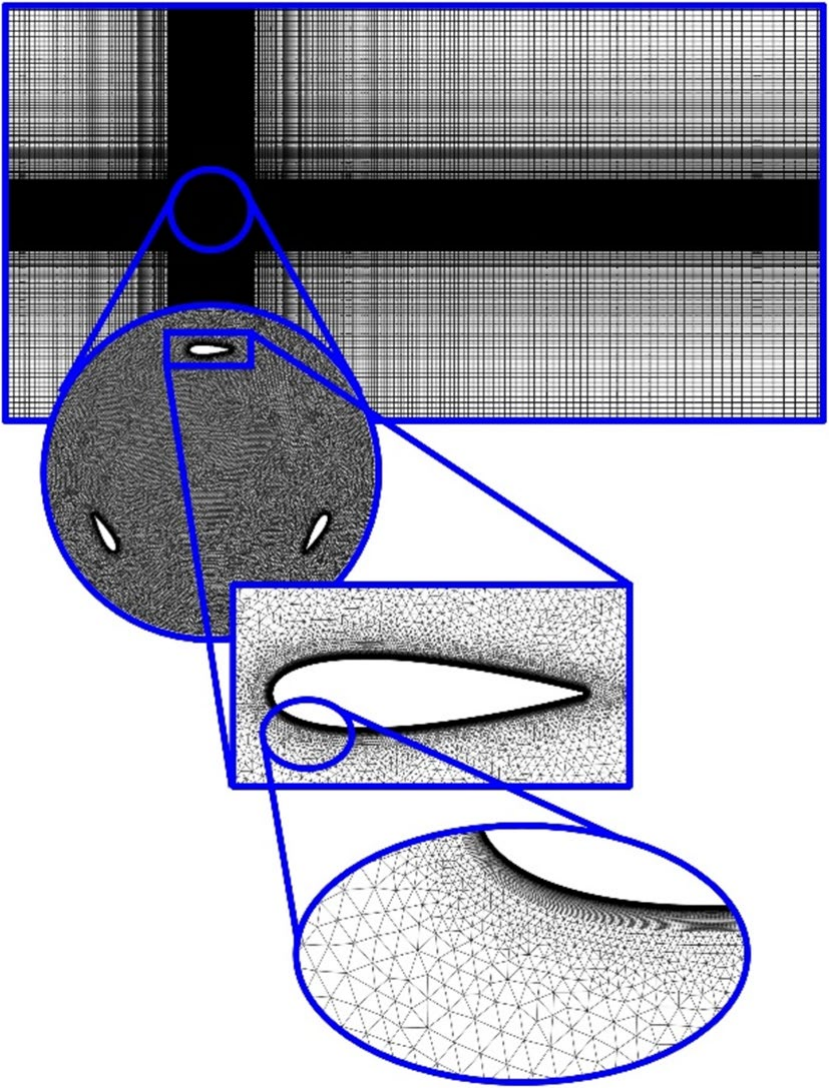
Computational domain mesh.

The computational domain, encompassing both static, and rotating parts, was meshed utilizing the ANSYS Meshing software. A structured mesh was employed, ensuring an appropriate boundary layer total thickness of $3\times {10}^{-3} m$ and a growth rate of 1.06. This mesh design adhered to the requirement of Y^+^ < 1, as specified by the $SST k-\omega$ turbulence model (Fig. 4).

**Figure 5:**
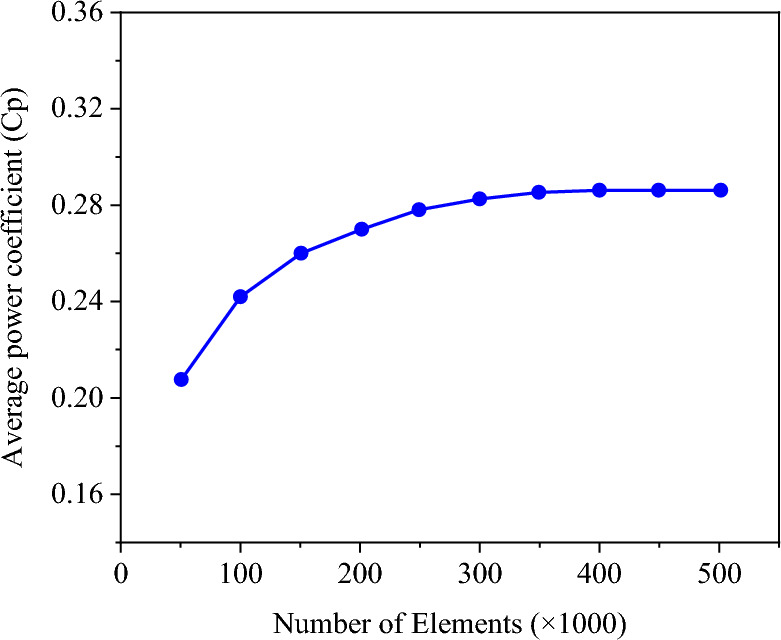
Mesh independence study.

**Figure 6:**
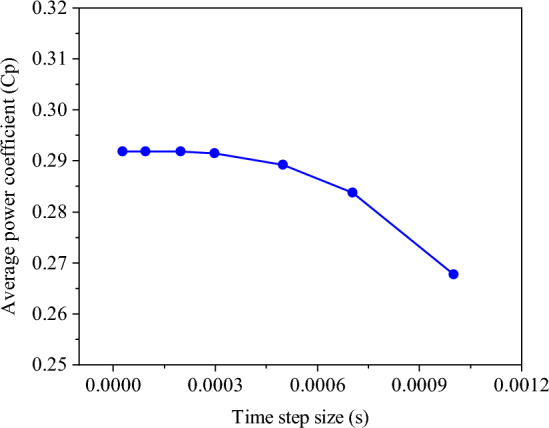
Time step independence study.

To assess mesh independence, the power coefficient was examined across a range of meshes, varying in density from 50,000 to 500,000 cells. Figure 5 illustrates that grids finer than 350,000 cells yield negligible changes in the results. Consequently, this particular mesh configuration was selected for subsequent simulations. Furthermore, the changes of ${\overline{C} }_{p}$ were investigated by decreasing the time step size from 0.003 to 0.0001 s (Fig. 6). It is evident from the figure that the ${\overline{C} }_{p}$ changes are minimal with decreasing the time step size after 0.0003 s. Therefore, A time step of 0.0003 s equivalent to (∆θ = 0.6$^\circ$) as the optimal value for this research is selected.

### Validation

To validate the numerical simulation results, experimental data published by Howell et al.^36^ were used. The investigated wind turbine was a 3-blade VAWT with a NACA0022 airfoil cross section. The chord length, blade height, and rotor diameter were 0.1 m, 0.4 m and 0.6 m, respectively. Validation analyzes were simulated according to Howell et al.’s conditions^36^ at a tip speed ratio of 2.45. The wind flow speed was 5.07 m/s, which resulted in a rotor rotational speed of 41.405 rad/s. More details are available in references^36,39^. The Reynolds number in the present research is equal to ${Re}_{D}=3.6\times {10}^{5}$.

**Figure 7:**
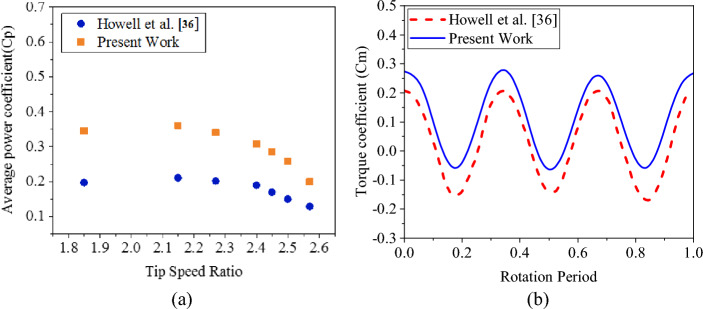
(**a**) Validation with the experimental data of Howell et al.^36^ (**b**)Validation of the Cm curve with the data of Howell et al.^36^.

In Fig. 7a, the average power coefficients in different tip speed ratios were compared with the 2D results and the experimental data of Howell et al.^36^. In Fig. 7b, the Cm curve with the data of Howell et al.^36^ were compared. It is evident that the 2D numerical simulation successfully captures the varying trend observed in the experimental curve. It was also able to predict the tip speed ratio of the maximum power coefficient accurately. Consequently, the 2D simulation can be deemed a reliable and valid alternative for the development of experimental research. The disparities between the 2D and 3D curves and experimental curves can be attributed to a set of factors. In experimental conditions, the length of the blades is limited and vortices are formed at both ends of the blades. Also, the friction of the surface and the arms causes a decrease in performance. The mentioned factors along with common simplifications in 2D simulation cause overestimation of results compared to experimental data. The numerical models used also have a significant impact on the results. Considering that the $SST k-\omega$ model is known as an efficient model in many studies, it is also used in the present study^9,30,37,39^. Overestimation of VAWT output power in 2D simulations is a common occurrence in numerical studies, as reported in papers by Howell et al.^36^, Benini et al.^37^, Wang et al.^13^, Zheng et al.^38^, and Li et al.^17^.

**Figure 8:**
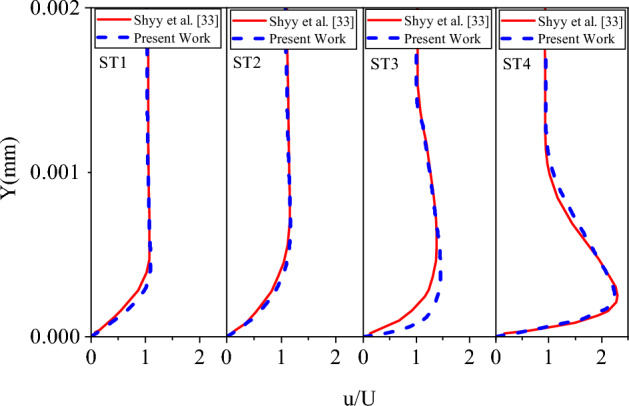
Validation of the results with the velocity profiles from Shyy et al.^33^.

Figure 8 shows a validation for the plasma actuator code. Based on Shyy et al.’s condition^33^, the actuator was simulated in 2D on a flat surface. In the mentioned figure, a comparison of the velocity profiles at four different sections with the data of Shyy et al.^33^ can be seen. A good agreement between the results, and experimental data is observed.

## Findings and discussions

### VAWT performance in baseline conditions

**Figure 9:**
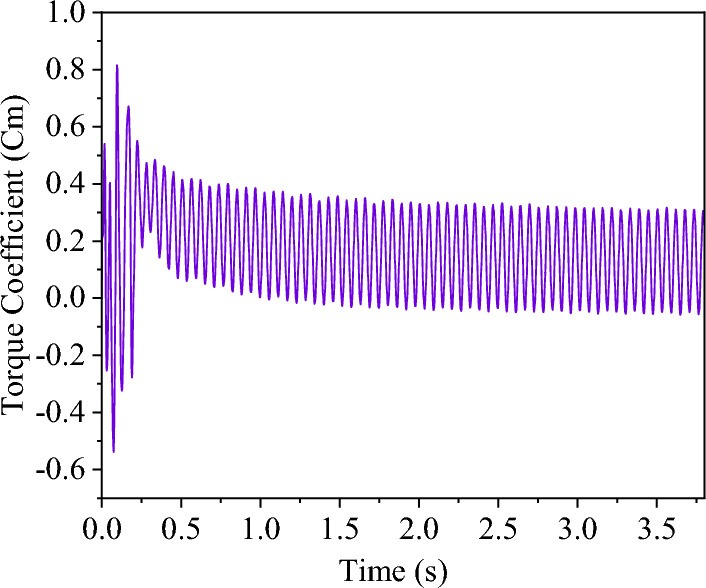
Wind turbine torque coefficient curve.

In order to study VAWT performance in baseline conditions, TSR = 2.15 was used, which leads to maximum ${\overline{C} }_{p}$. In Fig. 9, the ${C}_{m}$ curve of the turbine is shown. This curve covers a 3.8 s interval (22 rotation periods). About 2.6 s after the start of the simulation (15 rotation periods), the curve fluctuations take on a regular pattern.

**Figure 10:**
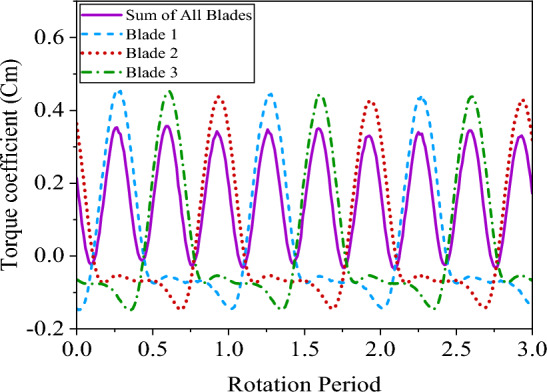
Cm curves for 3 rotation periods.

Figure 10 displays the torque coefficient curves for each blade as well as the sum of the blades during three rotation cycles. Each cycle includes a complete rotation of a blade from 0**˚** to 360**˚**. In baseline conditions, the power coefficient, production power, and production energy of VAWT were calculated as ${\overline{C} }_{p}=0.359$, ${P}_{Turbine}=3.44 W$, and ${E}_{Turbine}=0.595 J/cycle$, respectively. It should be noted that each turbine rotation period lasts $t=0.173s$.

The first half-cycle of a period (0° < θ < 180°) is known as upwind, in which a significant part of the torque is produced. Investigating the VAWT performance under the baseline conditions lead to finding the regions with the highest, and lowest torque coefficients. Examining the torque coefficient curve of a blade gives valuable information about how it changes. Around azimuth 0° negative torque intensification is observed owing to virtual camber that occurs when an airfoil is moving along a rotating path. In such a situation, a symmetrical airfoil is exposed to a curved stream. This is similar when a cambered airfoil is exposed to a straight stream. This causes torque and lift to be negative^19^. In the range of 30° to about 100°, an increase in the torque coefficient is observed. After 100°, flow separation intensifies, and dynamic stall occurs on the inboard edge of the airfoil. This causes a significant reduction in torque production. In these intervals, vortices are mainly formed on the inboard side of the blade. The second half-cycle of a period (0° < θ < 180°) is known as downwind, in which, the blade has a negative angle of attack, and vortices are mainly formed on the outboard edge of the airfoil.

### Influence of different force waveform

The focus of the present section is on investigating the effects of applying a plasma actuator with different force waveforms on the blades of a VAWT. Previous study conducted by Abbasi et al.^39^ examined the optimal actuator installation position. The study revealed that positioning the actuator on the inside edge of the airfoils, close to the leading edge, resulted in the most favorable performance. Positions were determined specifically for each blade to achieve the best outcomes. Based on these findings, in the present study this combined installation approach was employed. Various force waveforms were considered for the plasma actuator, and their results were compared to clean turbine. The first case examined involved the use of a plasma actuator with a constant force. In this scenario, the actuator remained active for the entire rotation period, applying a continuous body force to the boundary layer flow.

**Figure 11:**
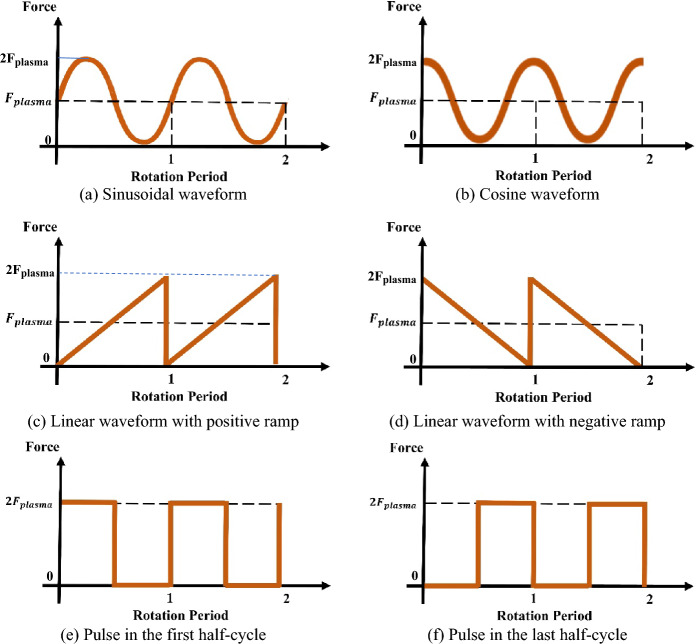
A summary of the investigated waveforms.

In the next step, two cases were examined, where the force was applied to the blades in a pulsed manner during the first, and last half-cycles of a rotation period. Subsequently, the plasma actuator force was explored with linear waveforms featuring positive and negative slopes. Specifically, the force was applied as periodic sawtooth functions, ascending and descending, within each rotation period of the rotor, with an amplitude of ${F}_{plasma}$. Additionally, sine and cosine waveforms were investigated, characterized by an amplitude of ${F}_{plasma}$, and a frequency equal to the rotational speed of the wind turbine. Consequently, over the course of a complete turbine rotation cycle, the plasma actuator force completed a full sine or cosine period. A summarized depiction of the investigated waveforms is presented in Fig. 11. The scaling of the waveforms was considered to have the same time-averaged plasma force as the steady state (the maximum value of all of them is ${2F}_{plasma}$); This approach was adopted in order to provide the possibility of comparing the impact of different waveforms and comparability of the results, including the power consumption of the actuator in each case. According to the following relationships, the amount of incoming forces caused by plasma in the center in the x direction is equal to ${F}_{x-Plasma}=$ $1.96\times {10}^{4}N$ and in the y direction is equal to ${F}_{y-Plasma}=9.78\times {10}^{3}N.$

**Figure 12:**
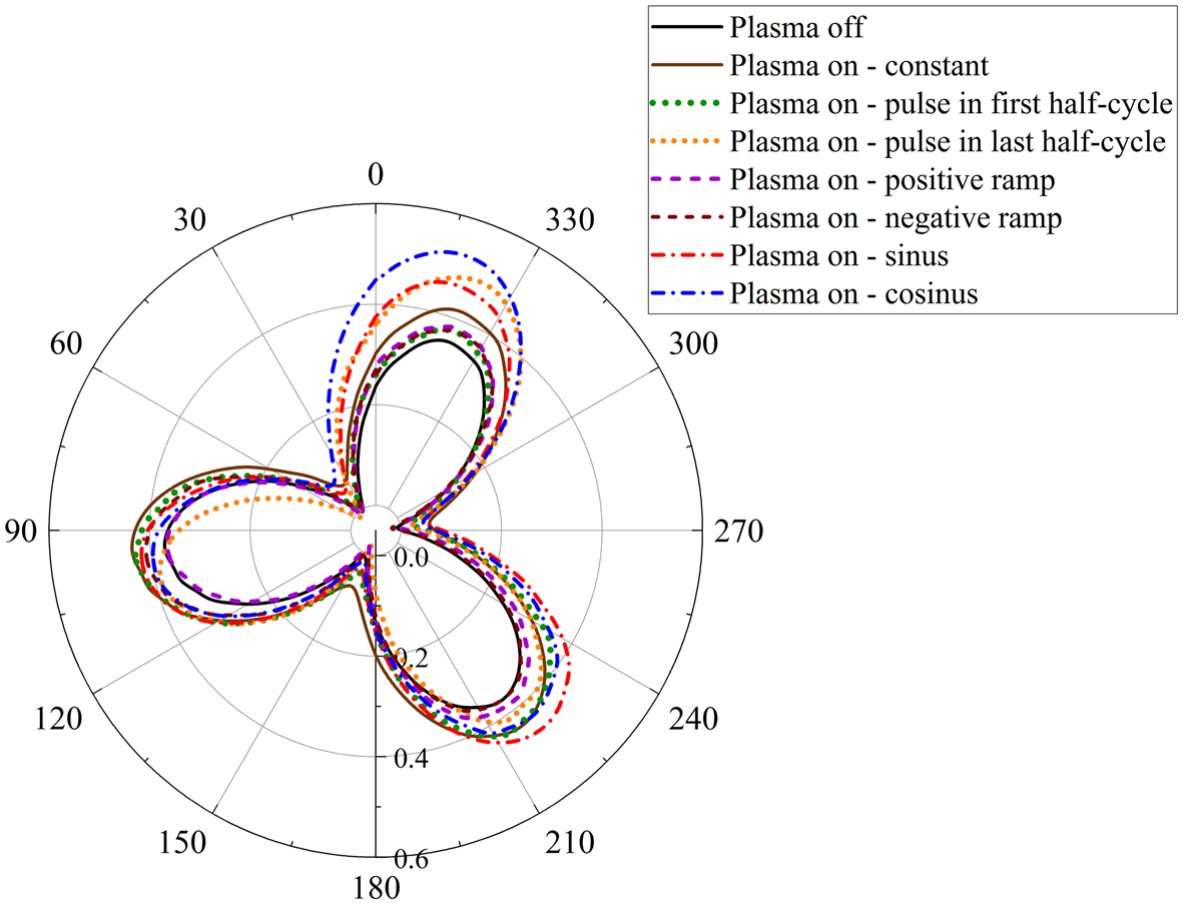
Comparison of the Cm curves of the different investigated cases.

The purpose is the matching of the beginning and end of the variable force cycles with the rotation cycle of each blade; Considering the phase difference between the three blades, it is obvious that for each blade, a dedicated code has been considered to apply the plasma actuator force. Figure 12 provides a comparison of the ${C}_{m}$ curves of the wind turbine for each examined case in comparison to the case without an actuator. Notably, the linear waveforms resulted in the least deviation in the torque coefficient curve. The constant force actuator exhibited a greater capacity to enhance the torque coefficient between azimuths ranging from 60° to 210°. Furthermore, the pulse waveform during the last half-cycle, and the pulse waveform during the first half-cycle demonstrated the potential to improve turbine performance, respectively. Among the examined waveforms, the cosine, and sinusoidal waveforms exhibited the highest rate of improvement. Specifically, the sinusoidal waveform outperformed other waveforms between azimuths of 90° to 270°. Conversely, the cosine waveform demonstrated performance enhancement at the beginning (0° to 30°), and end (240° to 360°) of the rotation cycle.

Table 2 provides the average ${\overline{C} }_{p}$ for the investigated cases, aiming to provide an accurate assessment of the various waveforms. Additionally, the comparison of ${\overline{C} }_{p}$ percentage changes with the baseline case is presented. The results reveal that the cosine waveform exhibits the highest enhancement, raising the VAWT power coefficient to 0.548. This represents a substantial increase of 52.64% compared to the baseline case. Conversely, the positive ramp waveform demonstrates the lowest increase, with a modest improvement of 14.48%.

**Table 2:** Comparison of the ${\overline{C} }_{p}$ of the different investigated cases.

	${\overline{C} }_{p}$	Changes in ${\overline{C} }_{p}$ (%)
Plasma off	0.359	–
Constant force	0.475	32.31
Pulse in first half-cycle	0.480	33.70
Pulse in last half-cycle	0.465	29.53
Positive ramp	0.411	14.48
Negative ramp	0.424	18.10
Sine waveform	0.542	50.97
Cosine waveform	0.548	52.64

**Figure 13:**
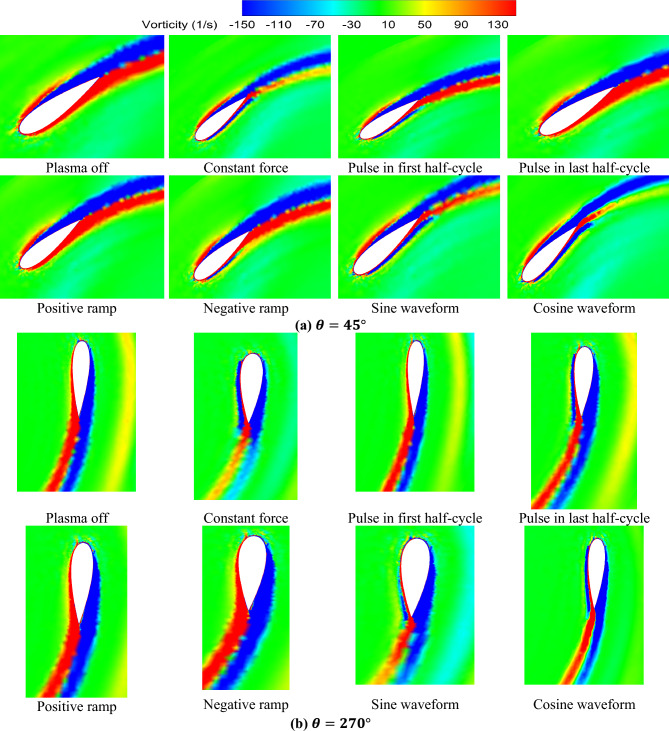
Comparison of the vorticity contours.

Figure 13 illustrates the vorticity contours for the examined cases in comparison to the baseline case. These contours are presented at two specific azimuths, namely 45°, and 270°, which serve as representatives of upwind, and downwind rotations, respectively. It is evident from the figure that the application of the plasma actuator with the cosine force waveform leads to a significant reduction in vorticity for both upwind, and downwind rotations, surpassing the effects observed in other cases. Notably, the reduction in vorticity is particularly pronounced during the downwind rotation when the sinusoidal waveform is employed. The constant force actuator also demonstrates effectiveness in reducing vorticity, albeit to a lesser extent compared to the aforementioned cases. On the other hand, the remaining waveforms exhibit a relatively lesser impact on flow separation control, and vortices reduction.

**Figure 14:**
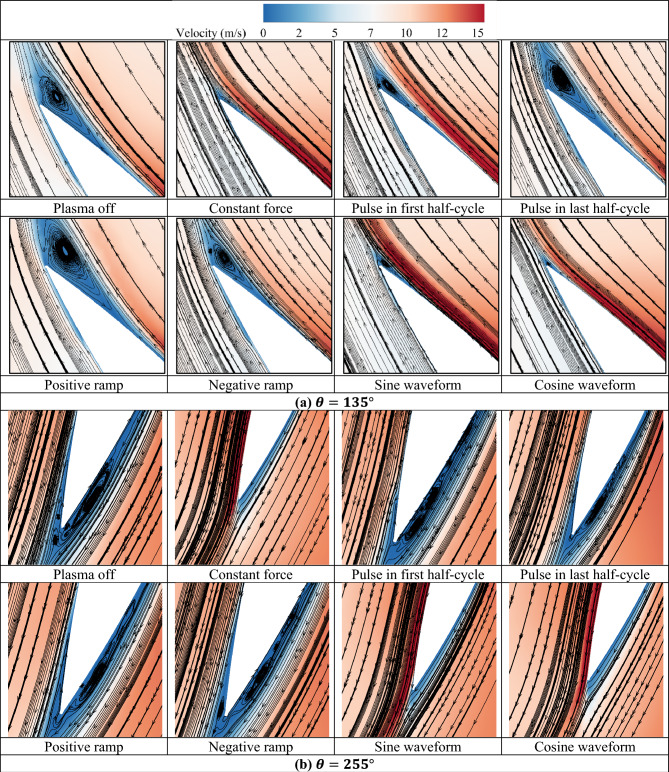
Relative velocity contours with streamlines on blades.

Figure 14 shows the relative velocity contours and streamlines for the different investigated waveforms. The streamlines are displayed on the inboard side of the blade at the azimuth of $135^\circ$. This azimuth was chosen as an example of areas prone to flow separation. As can be seen, the cosine and sinusoidal waveforms had the greatest impact and the pulse and linear waveforms had the least impact on the control and delay of flow separation.

**Figure 15:**
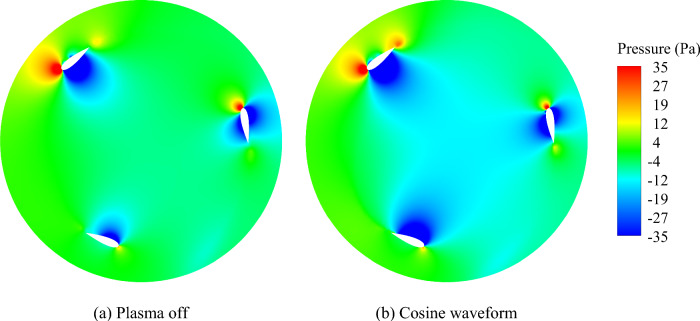
Comparison of the Pressure contours.

**Figure 16:**
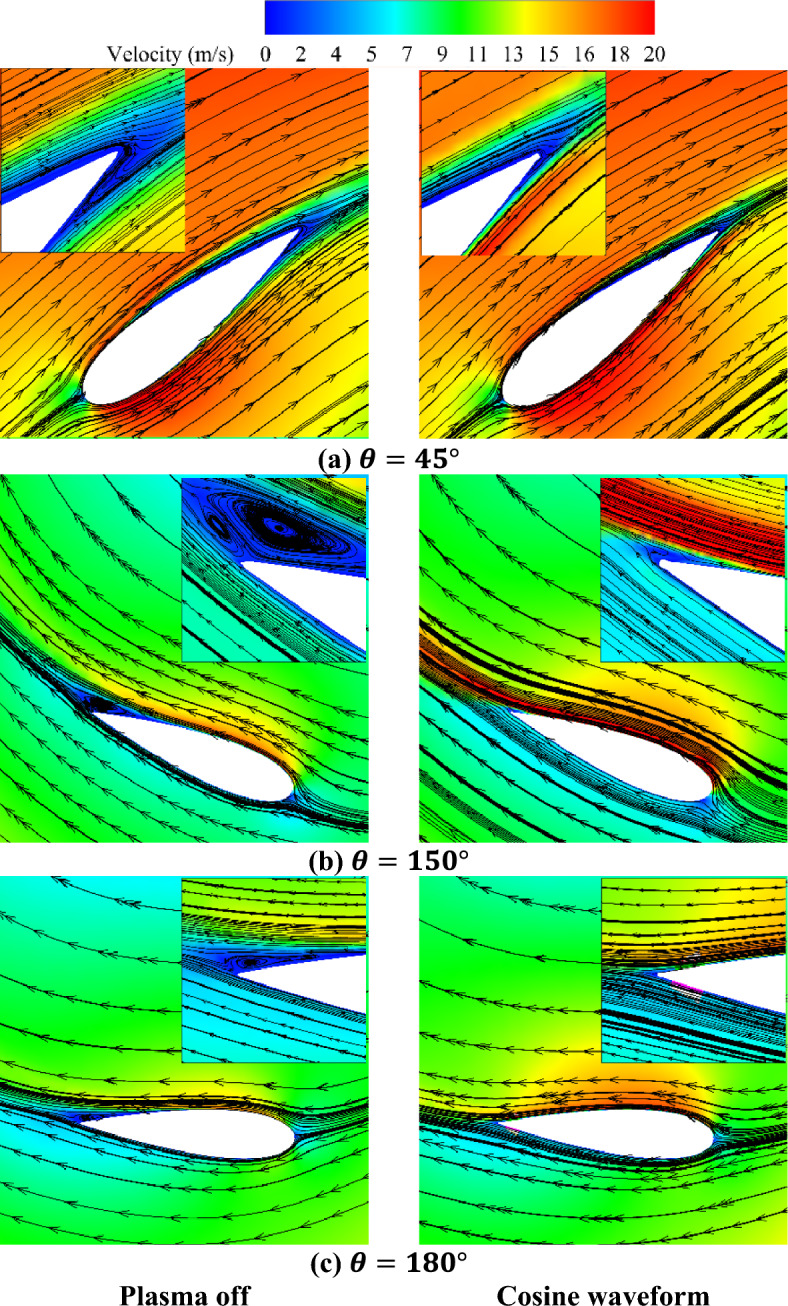
Comparison of the velocity contours (**a**) Plasma off, (**b**) Cosine waveform.

Figures 15 and 16 depict the static pressure and velocity contours on the VAWT blades for the cosine waveform, which emerged as the most favorable case compared to the baseline.

The actuator, through the injection of momentum into the flow, leads to an augmentation of the boundary layer velocity, reducing flow separation, and suppressing vortices. This, leads to an enhancement in VAWT performance. The comparison between the cosine waveform and the baseline cases reveals several key observations. Firstly, using plasma actuator to control the flow on the inside edge of the airfoils amplifies the local velocity while concurrently diminishing the local static pressure compared to the baseline state. Furthermore, Fig. 16 illustrates the streamlines, indicating that the induction of force by the actuator results in a delay in the onset of flow separation. These findings collectively demonstrate the positive impact of the plasma actuator, specifically with the cosine waveform, on the VAWT performance. The actuator ability to increase local velocity, reduce local static pressure, and delay flow separation contributes to the overall improvement in the turbine's efficiency, and effectiveness.

**Figure 17:**
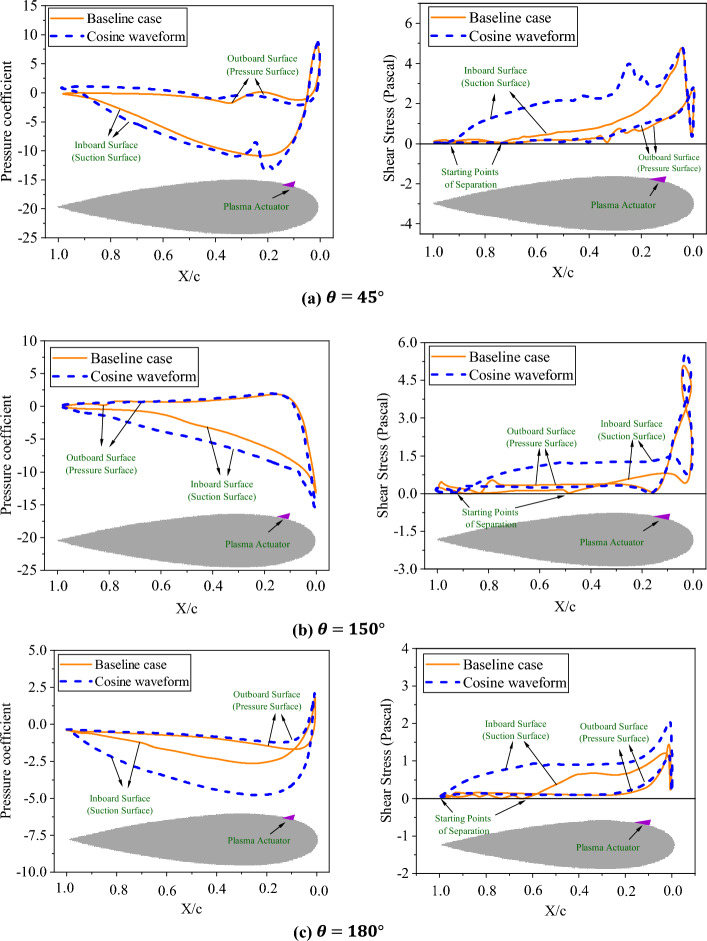
Comparison of the pressure coefficient and shear stress in baseline and controlled cases.

Figure 17 shows the changes of shear stress and pressure coefficient of a blade in the azimuths of 45°, 150°, and 180°. The blade cross-section profile is depicted below the curves. These azimuth positions were selected as areas with the highest amount of vortices formation, and greater potential for flow separation.

Based on Fig. 17, it can be seen that using actuators reduced the pressure on the inner edge of the airfoil, which is owing to the increase in the velocity of the boundary layer due to the body force effects. The significant pressure difference on the two edges of the airfoil increases lift and torque. Analysis of the shear stress on the blade surface provides the possibility of finding the exact points of flow separation. It can be seen that the utilization of the actuator causes the separation to start with a significant delay compared to the baseline state. In the baseline case, the shear stress reaches zero at approximately 75% c, 50% c, and 60% c for azimuths of 45°, 150°, and 180°, respectively. However, with the implementation of the body force, it increases, and delays the occurrence of flow separation to approximately 90% c, 85% c, and the trailing edge for azimuths of 45°, 150°, and 180°, respectively.

### Plasma actuator power consumption

To evaluate the cost-effectiveness of using actuator, it is necessary to determine the net energy generated by VAWT after accounting for the energy consumed by the plasma actuators. Table 3 provides the average power coefficients of the VAWT for different investigated cases. Using the correlations, and relationships established by Shyy et al.^33^ and Yoon et al.^40^, the actuator power consumption in the case with a constant force was calculated as ${P}_{Actuator}=1.112 W$. This allows us to calculate the power that each of the actuators consumes during one complete rotation period as ${E}_{Actuator}=0.192 J/Cycle$. Consequently, the net energy output of the wind turbine is determined according to Eq. (13).

13 $${E}_{Net}={E}_{Turbine}-{E}_{Actuators},$$

where ${E}_{Net}$ represents the net energy of the VAWT, ${E}_{Turbine}$ denotes the total generated energy by VAWT (mentioned in Sect. “VAWT performance in baseline conditions”), and ${E}_{Actuators}$ is the total energy consumed by the plasma actuators.

**Table 3:** Comparison of the ${\overline{C} }_{p}$ of the different investigated cases.

	${\overline{C} }_{p}$	Changes in ${\overline{C} }_{p}$ (%)	*E (J/cycle)*		Changes in ${E}_{net}$ (%)
			${E}_{Turbine}$	${E}_{net}$	
Plasma off	0.359	–	0.59	0.59	–
Constant force	0.475	32.31	0.78	0.20	− 65.42
Pulse in first half-cycle	0.480	33.70	0.80	0.50	− 15.25
Pulse in last half-cycle	0.465	29.53	0.77	0.47	− 20.34
Positive ramp	0.411	14.48	0.68	0.38	− 35.59
Negative ramp	0.424	18.10	0.71	0.41	− 30.50
Sine waveform	0.542	50.97	0.90	0.80	35.59
Cosine waveform	0.548	52.64	0.91	0.81	37.28

**Figure 18:**
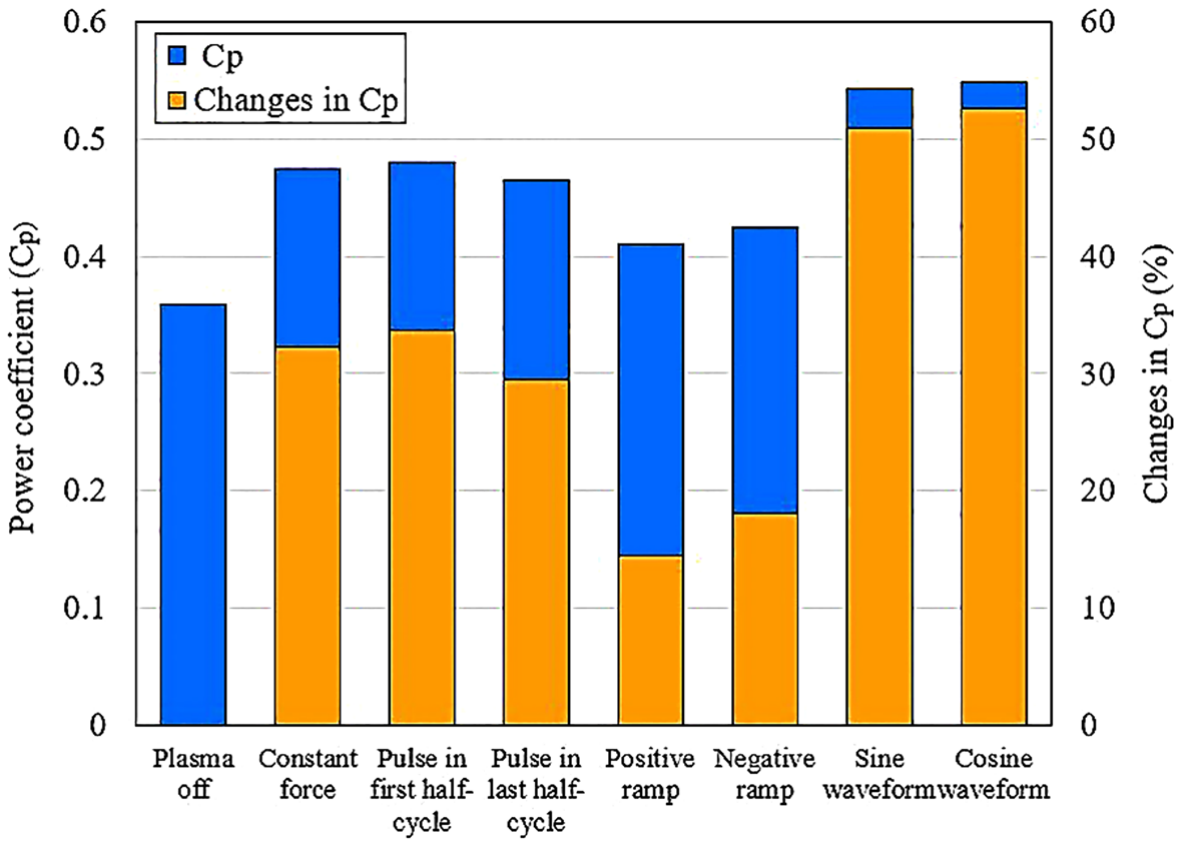
The effect of different waveforms on VAWT power coefficient.

**Figure 19:**
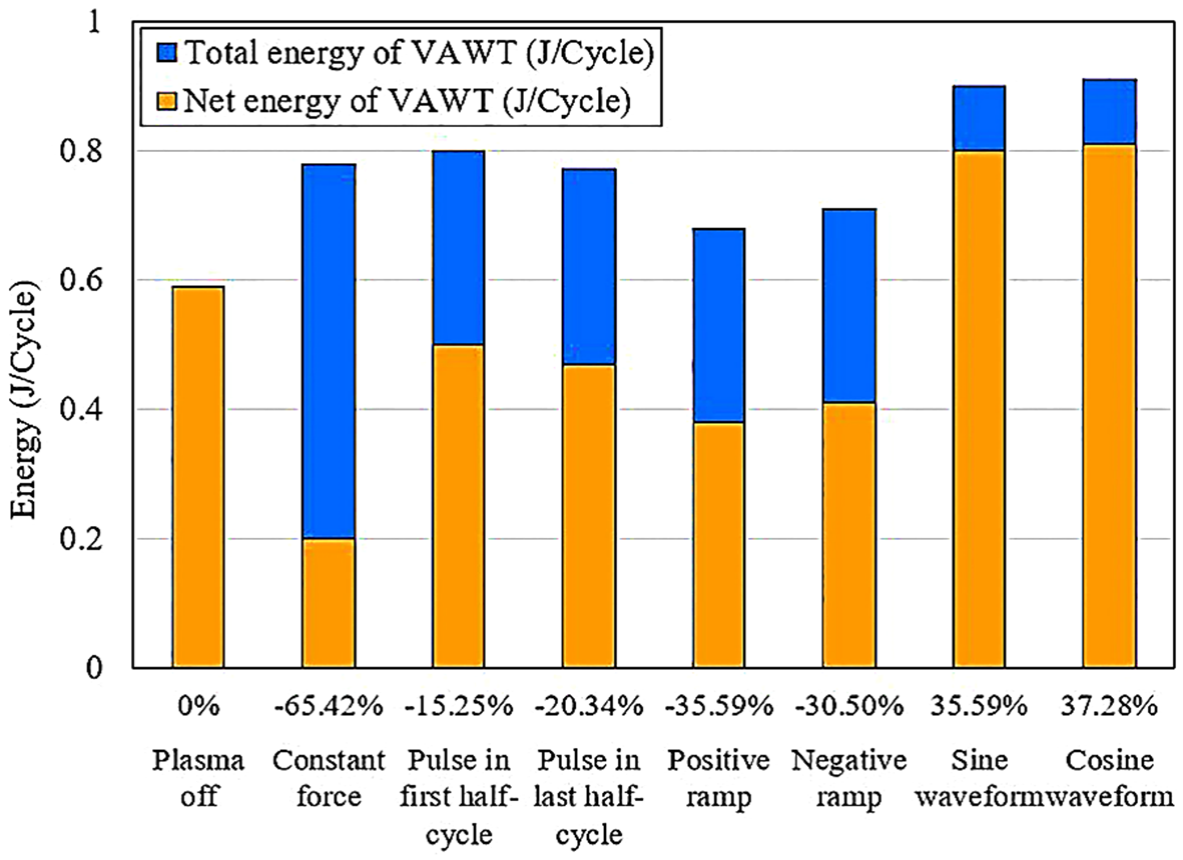
The effect of different waveforms on the total and net energy of VAWT.

Table 3 demonstrates that the cosine waveform exhibited the highest performance, resulting in a 52.64% ${\overline{C} }_{p}$ increase in comparison to the plasma off case. However, considering the cost of energy consumption of control equipment, in order to determine the feasibility of using the actuator, is crucial. Therefore, in Table 3 the percentage changes of ${E}_{Net}$ are presented in comparison to the plasma off case. In Fig. 18, the changes of average power coefficient of VAWT under different strategies are shown. Also, the percentage of ${\overline{C} }_{p}$ changes compared to the baseline case for each waveform is presented. Figure 19 compares the net energy produced by VAWT with the total energy, considering the energy consumed by the actuator. In addition, the percentage of changes compared to the baseline case is provided for each case. It is evident that in most cases, the energy consumption outweighed the benefits. The cosine and sinusoidal waveforms, even after accounting for the actuator energy consumption, still showed the most significant improvements with a 37.28% and 35.59% increase, respectively, compared to the baseline case.

Examining the data in Table 4 allows comparing the improvement caused by the optimal case (cosine waveform) with the findings from other studies. The aim of this comparison is to examine the advantages of employing a time-varying force in contrast to a constant force generated by the plasma actuator. It should be noted that each of these studies has different conditions, and characteristics, and this comparison solely highlights the effectiveness of the method proposed in this research compared to other approaches. The results demonstrate that the utilization of variable force actuators, as opposed to simple actuators, exhibits superior capability in controlling flow behavior, and enhancing the VAWT performance. Thus, it can be regarded as an impactful, and effective method.

**Table 4:** The results of the present work in comparison with previous research.

Study	Relative increase in performance (%)	Study	Relative increase in performance (%)
Present work	37.28	Nakano et al.^21^	25.1
Greenblatt et al.^12^	38	Benmoussa et al.^14^	11
Ma et al.^13^	34.3	Xu et al.^17^	43.836
Pescini et al.^22^	25.5	Jafari et al.^18^	28
Matsunuma et al.^25^	38	Chavoshi et al.^19^	10

## Conclusion

The performance of a Darrieus straight-blade VAWT was modeled and analyzed with ANSYS Fluent. Assessing the impact of the plasma actuator with time-varying force on the flow behavior on the blades of a VAWT is the main objective of the present work. Different force waveforms, such as constant, sine, cosine, positive ramp, negative ramp, pulse in the first half-cycle, and pulse in the last half-cycle, were applied to the wind turbine blades using the plasma actuator, allowing for a comprehensive investigation of their effects. The findings indicate that:

1. Linear force waveforms caused the least change in the ${C}_{m}$ curve (14.48% increase in ${\overline{C} }_{p}$). The actuator with constant force had a greater ability to increase the torque coefficient compared to linear waveforms, especially between azimuths from 60**˚** to 210**°**.
2. The sinusoidal waveform could perform better than other curves between the azimuths from 90**°** to 270**°**. In general, it improved the ${\overline{C} }_{p}$ of VAWT by 50.97%.
3. The highest rate of improvement belonged to cosine waveform by 52.64%. It improved the torque coefficient at the beginning of the rotation cycle (from 0**°** to 30**°**), and at the end (from 240**°** to 360**°**).
4. The cosine and sinusoidal waveforms, after deducting the actuator energy consumption, led to the greatest improvement with 37.28% and 35.59% increase, respectively, compared to the baseline case.
5. Applying the time-varying force is a more effective way to use the plasma actuator. It provides the possibility of significant improvement of VAWT performance in comparison to similar methods.

## Data Availability

The datasets used and/or analyzed during the current study available from the corresponding author on reasonable request.
